# POOLING COHORTS ACROSS THE LIFE COURSE TO IDENTIFY NOVEL EARLY-LIFE DETERMINANTS OF HEALTH OUTCOMES IN LATER-LIFE

**DOI:** 10.1093/geroni/igae098.0531

**Published:** 2024-12-31

**Authors:** Katrina Kezios, Scott Zimmerman, Peter Buto, M Maria Glymour, Adina Zeki Al Hazzouri

**Affiliations:** Columbia University, New York, New York, United States; University of California San Francisco, San Francisco, California, United States; University of California San Francisco, San Francisco, California, United States; Boston University, Boston, Massachusetts, United States; Columbia University, New York, New York, United States

## Abstract

To answer novel questions about exposure-disease effects over the entire life course, researchers are increasingly pooling data from multiple cohorts covering different life periods to construct complete life course data. If carefully constructed, these “synthetic cohorts” should enable rigorous investigation of earlier-life determinants and pathways to health in older age. Using simulations, we previously demonstrated a matching-based approach to create a synthetic cohort where individuals with exposure (“A”) information in an early-life cohort were matched to individuals with outcome (“Y”) information in a later-life cohort based on mediators and confounders of A and Y. Here, we describe results from a proof-of-concept analysis using a sample-splitting approach in the Health and Retirement Study (1994-2018). We examined the relationships between BMI in 1994 and 1) general health, 2) diabetes, and 3) systolic blood pressure in 2018 in synthetic cohorts constructed using 2008 as the matching time point. Synthetic cohort estimates were benchmarked against those in the longitudinal HRS sample observed from 1994-2018. In the proof-of-concept analyses, our approach was most successful when a later measure of the exposure and/or earlier measure of the outcome was available in both cohorts at the matching time-point. For example, for BMI1994 and general health2018, matching cohorts on BMI and general health in 2008 alone produced an estimate of -0.0386 [-0.054, -0.023] in the synthetic cohort (compared with -0.0334 [-0.040, -0.027] in the full cohort). We use these findings to guide analyses examining the relationship between other early life social and cardiovascular determinants and later-life health.

